# Fermentation, Infection and Immunity

**Published:** 1893-06

**Authors:** 


### Book Notices.

FERMENTATION, INFECTION AND IMMUNITY: A New Theory of these Processes which unifies their Primary Causation and places the Explanation of their Phenomena in Chemistry, Biology and the Dynamics of Moleculur Physics. By J. W. McLaughlin, M. D. Austin, Texas: Eugene Von Boeckmann, 1892.

In this work we have evidence that the processes of fermentation, infection and immunity are exciting as much interest amongst our cousins in America as they are on this side of the Atlantic. The author writes with the special object of showing "that the accepted principles of molecular physics and those of chemistry and biology, if supplemented by legitimate deductions from them, are amply sufficient to account for all the known phenomena of these processes and also to explain their relationship and intimate nature." In this Dr. McLaughlin cannot be accused of want of hopefulness, and many will feel inclined to criticise this predominant feature of his work. As, however, he shows that he has not only carefully studied his subject, but has also devoted a considerable amount of thought to some of the points he has taken up, his words are worthy of attention, as they show in what direction the thoughts of an observer who is not actually engaged in the experimental proof of the facts that he adduces, are likely to run. After going over the various theories of immunities and giving them fairly and fully, he then puts forward his theory "why the wave motions of a ferment and a fermentable substance must recur in a relative order of time, that those of the first can disrupt and convert those of the second into ferment products, likewise the wave motions of a bacterium and those of ultimate albuminoid molecules must recur in equal periods that the bacterium can convert the albuminoid molecules into poisonsus albumens; the products in both cases, it will be remembered, give off wave motions that interfere with those of the respective bacteria and in this way inhibit their action." Outside the body in artificial media this may be simple enough, but within the body, where the question of immunity has to be considered, it must be borne in mind that there is great diversity in the molecular structure of the various closely allied albumens, and Dr. McLaughlin hold that here "a dynamic change in any single group of these bodies may induce change in other groups, and that the products of such changes may in some cases be harmless and in others harmful." He then works out the process of the conversion of susceptible into "immune" albuminoids and speaks of the whole matter of immunity as "being resolved into the question of susceptibility or non-susceptibility of the albuminoid molecules of the animal body to molecular bombardment of a given bacterium." It is somewhat difficult to follow Dr. McLaughlin in his descriptions and especially to see that he offers anything like proof of his physical theory; nevertheless we can recommend this as an interesting philosophical disquisition, which, however theoretical it may be, and however much we may disagree with certain of the author's deductions and inductions, affords some food for thought,—*London Lancet*, April 29, '93, p. 1002.

